# Multiple integration of the gene *ganA* into the *Bacillus subtilis* chromosome for enhanced β-galactosidase production using the CRISPR/Cas9 system

**DOI:** 10.1186/s13568-019-0884-4

**Published:** 2019-09-30

**Authors:** Hildegard Watzlawick, Josef Altenbuchner

**Affiliations:** Institute of Industrial Genetics, University of Stuttgart, Allmandring 31, 70569 Stuttgart, Germany

**Keywords:** *Bacillus subtilis*, Multiple integration, CRISPR–Cas9 genome editing, *Bacillus subtilis* β-galactosidase

## Abstract

The *ganA* gene from *Bacillus subtilis* encoding a β-galactosidase for degradation of the galactomannan was integrated in different loci of the *B. subtilis* chromosome employing the CRISPR/Cas9 system. Hereby a total of five copies of *ganA* cassettes in which the *ganA* gene was fused with the glucitol-promoter were inserted in the recipient chromosome wherein hypothetical, sporulation and protease genes were deleted. The strain with five copies of *ganA* expression cassette showed a β-galactosidase activity similar to the one with the same gene on a pUB110 derived multi-copy plasmid and under the same regulatory control of the glucitol promoter and GutR activator. The production of β-galactosidase in the strain with the multi-copy plasmid decreased rapidly when growth was performed under induced conditions and without antibiotic selection. In contrast, the strain with the five copies of *ganA* in the chromosome produced β-galactosidase for at least 40 generations. This demonstrates that the CRISPR/Cas9 system is a valuable and easy tool for constructing stable producer strains. The bigger efforts that are needed for the multiple target gene integration into the chromosome compared to cloning in expression vectors were justified by the higher stability of the target genes and the lack of antibiotic resistance genes.

## Introduction

The production of recombinant proteins in high yield and high quality is a major task in biotechnology. Hereby, bacterial expression systems (Terpe 2006) are attractive because of the rapid cell growth to high cell densities in inexpensive media. The most commonly used host is *E. coli* due to their easy handling, the wealth of knowledge and the large repertoire of expression vectors. Other important industrial microorganisms are *Bacilli*. More than one-third of industrial enzymes are produced by *Bacillus* strains (Meissner et al. 2015). For heterologous gene expression the Gram-positive *B. subtilis* has a long history of industrial use and is widely appreciated as a cell factory (Cai et al. 2019; Jeong et al. 1998; Liu et al. 2013; Schallmey et al. 2004). It is regarded as “Generally Recognized As Safe” (GRAS) for its lack of endotoxins and is used as a food-grade expression host (de Boer Sietske and Diderichsen 1991). Under stress conditions cells develop a competence status which allows highly efficient uptake of DNA for genetic engineering (Chen and Dubnau 2004; Claverys et al. 2006; Kramer et al. 2007). There are two strategies available for overproduction of recombinant proteins, one is the cloning of the target gene into expression vectors and the other one is to integrate the target gene under a strong promoter into the chromosome. There is a large repertoire of vectors available and the use of high copy vectors ensures high protein production (Brockmeier et al. 2006; Heravi et al. 2015; Schumann 2007; Xia et al. 2007). A drawback of these vectors, based mostly on rolling circle replication is their structural and segregation instability (Fleming and Patching 1994; Shoham and Demain 1991) and the need of antibiotics for selection of plasmid maintenance. The integration of the target gene into the chromosome ensures high stability but due to the single copy of the gene gives less yield of product (Janniere et al. 1985; Mori et al. 1988; Motejadded and Altenbuchner 2007). This limitation can be overcome by integration of tandemly amplified target genes or by integration of multiple copies of the target gene at various positions of the chromosome (Huang et al. 2017; Kiel et al. 1995; Petit et al. 1990, 1992; Slugeňová et al. 1993; van der Laan et al. 1991; Wang et al. 2004; Yomantas et al. 2011; Young 1984). Tandemly amplified copies of genes are unstable as well (Petit et al. 1992; van der Laan et al. 1991; Young 1984), although there are other studies reporting high stability of the amplicons (Janniere et al. 1985; Vázquez-Cruz et al. 1996). Nevertheless, the integration of genes at multiple loci is the most promising way to achieve high yield and stable productivity. The antibiotic resistance genes necessary for selection of the integration events can be removed either by site-specific recombination (Bloor and Cranenburgh 2006; Sanchez et al. 2007; Sanchis et al. 1997) or avoided by using markerless systems (Kostner et al. 2017; Wenzel and Altenbuchner 2015; Zakataeva et al. 2010; Zhang et al. 2006). The Clustered, Regular Interspaced Short Palindromic Repeat (CRISPR) system with Cas9 as targeted nuclease was recently adapted for its use in *B. subtilis* (Altenbuchner 2016; Burby and Simmons 2017; Hong et al. 2018; Westbrook et al. 2016) and speculated to be ideal for multiple gene integration into the *B. subtilis* chromosome. It allows precise deletions and insertions without leaving behind antibiotic resistance genes and other scares such as recognition sites for site-specific recombinases. Irrespective of the methods used for target integration another important question to consider are the integration sites. They should not influence growth and protein production. In an attempt to reduce the *B. subtilis* genome it has been shown that there are hundreds of genes and large chromosomal regions which can be deleted without a negative influence on cell growth (Aguilar Suárez et al. 2019; Commichau et al. 2013; Manabe et al. 2012, 2013; Morimoto et al. 2008; Reuß et al. 2017; Tanaka et al. 2013). Concomitant deletions and insertions even offer the possibility to remove unwanted gene products like proteases and properties like sporulation or prophages.

As a proof of principle we integrated the *ganA* gene from *B. subtilis* encoding a β-galactosidase for degradation of the galactomannan (Watzlawick et al. 2016) five times into the *B. subtilis* chromosome and hereby deleted various hypothetical, sporulation and protease genes. Herby the *ganA* gene was fused with the glucitol promoter (Poon et al. 2001; Ye et al. 1994; Ye and Wong 1994) to generate a *Pgut*-*ganA* expression cassette. The strain with five copies of the *ganA* expression cassette showed a β-galactosidase activity similar to one with the same gene on a pUB110 derived multi-copy plasmid. In contrast to the multi-copy plasmid the β-galactosidase activity was stable for many generations even under induced conditions whereas the multi-copy plasmid was rapidly lost. This demonstrates that the CRISPR/Cas9 system is an efficient method to introduce genes multiple times into a bacterial chromosome for high and stable protein production.

## Materials and methods

### Strains, media, and growth conditions

*Escherichia coli* JM109 (Table 1) was used for plasmid propagation and gene expression throughout this study. *E. coli* strains containing the desired plasmids were selected on Luria–Bertani media (LB) plates supplemented with ampicillin (100 µg/ml) or kanamycin (50 µg/ml) depending on the plasmid selection marker. For blue/white screening IPTG (50 µg/ml) and X-Gal (80 µg/ml) was added. *B. subtilis* cells were grown on LB plates as well, for selection of plasmids kanamycin was added at 5 µg/ml. *B. subtilis* REG19 is derived from *B. subtilis* 168 and contains an additional copy of the *comK* and *comS* gene under control of the *B. subtilis* mannitol promoter (Rahmer et al. 2015). Step by step, five different gene deletions and concomitant insertions of *ganA* under control of the *gut* promoter *P*_*gut*_, were introduced in REG19 to obtain the strains JA-Bs34, JA-Bs35, JA-Bs36, JA-Bs37 and JA-Bs40 (see Table 1). For induction of *ganA* expression, the overnight cultures in LB with 0.5% (w/v) glucose were diluted to 0.05 OD_600_ in 10 ml LB with 0.5% (w/v) glucitol and for repression of *ganA* strains were grown in LB with 0.5% glucose. The cells were incubated in shake flasks at 37 °C and 200 rpm on a shaking platform. In case of REG19/pHWG1132 kanamycin was supplemented in the medium for the preculture and during induction. After 16 h the cells were harvested by centrifugation.

**Table 1:** Strains used in this study

Strain or plasmid	Genotype or relevant structure	Source or references
*E. coli*		
JM109	*rec*A1, *end*A1, *gyr*A96, *thi*-1, *hsd*R17(r_K_^−^, m_k_^+^), *mcr*A, *sup*E44, *gyr*A96, *rel*A1, λ^−^, Δ(*lac*-*proAB*), F’ (traD36, *pro*AB + , *lac*I^q^, (Δ*lacZ*)M15)	Yanisch-Perron et al. (1985)
*B. subtilis*		
Reg19	*trpC2, ΔmanPA::ermC, P*_*mtlA*_-*comK*-*comS*	Rahmer et al. (2015)
JA-Bs34	*trpC2, ΔmanPA::ermC, P*_*mtlA*_*comK*-*comS, Δ(spoIISA, spoIISB)::P*_*gut*_-*ganA*	This study
JA-Bs35	*trpC2, ΔmanPA::ermC, P*_*mtlA*_-*comK*-*comS, Δ(spoIIA, spoIIB)::P*_*gut*_-*ganA, Δ(cotA, cotZ, cotY, cotX, cotW, cotC, yjcA, yjcK, yjcZ, spoVIB)::P*_*gut*_-*ganA)*	This study
JA-Bs36	*trpC2, ΔmanPA::ermC, P*_*mtlA*_-*comK*-*comS, Δ(spoIIA, spoIIB)::Pgut*-*ganA, Δ(cotA, cotZ, cotY, cotX, cotW, cotC, yjcA, yjcK, yjcZ, spoVIB)::P*_*gut*_-*ganA), ΔnprB*:*:P*_*gut*_-*ganA*	This study
JA-Bs37	*trpC2, ΔmanPA::ermC, P*_*mtlA*_-*comK*-*comS, Δ(spoIIA, spoIIB)::Pgut*-*ganA, Δ(cotA, cotZ, cotY, cotX, cotW, cotC, yjcA, yjcK, yjcZ, spoVIB)::P*_*gut*_-*ganA), ΔnprB*:*:P*_*gut*_-*ganA, Δ(ywrJ, cotB, cotH, cotG, ywrF, ywrE)*:*:P*_*gut*_-*ganA*	This study
JA-Bs40	*trpC2, ΔmanPA::ermC, P*_*mtlA*_-*comK*-*comS, Δ(spoIIA, spoIIB)::Pgut*-*ganA, Δ(cotA, cotZ, cotY, cotX, cotW, cotC, yjcA, yjcK, yjcZ, spoVIB)::P*_*gut*_-*ganA), ΔnprB*:*:P*_*gut*_-*ganA, Δ(ywrJ, cotB, cotH, cotG, ywrF, ywrE)*:*:P*_*gut*_-*ganA, ΔamyE*:*:P*_*gut*_-*ganA*	This study

### Construction of plasmids

**Fig. 1:**
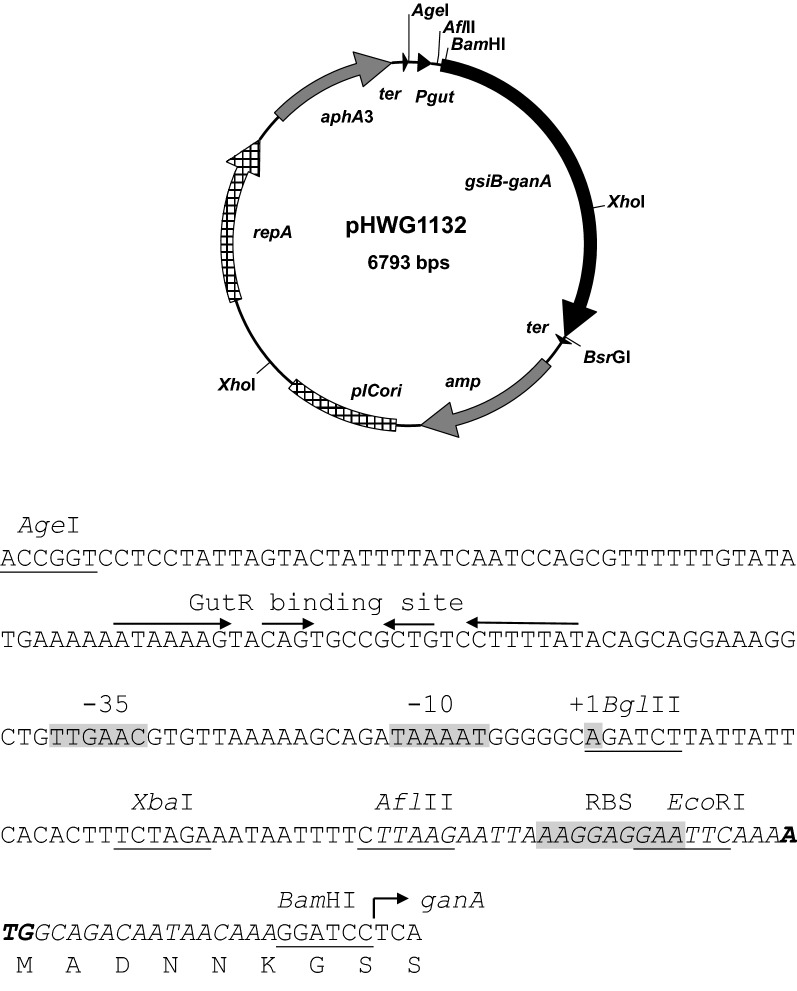
Restriction map of the expression vector pHWG1132 and the transcription and translation initiation region of *ganA* consisting of the *gut* promoter with the GutR binding site (arrows) and the − 35, − 10 and + 1 sequence (grey blocks). The sequence derived from *gsiB* is shown in italics, the ATG start codon in bold letters and restriction sites are underlined. The sequence between the *Bgl*II site and *Afl*II site is derived from vector pJOE5751.1

The plasmid pJOE8386.1 is a shuttle vector including the origin of replication and the kanamycin resistance gene of pUB110 (Jalanko et al. 1981) the origin and the ampicillin resistance of pIC20HE (Altenbuchner et al. 1992). The plasmid carries an expression cassette consisting of an enhanced green fluorescent protein (eGFP) gene fused with the translation initiation region of *gsiB* (Wenzel et al. 2011) and the *gut* promoter, both from *B. subtilis*. In addition, Rho-independent transcription terminators were introduced upstream of the *gut* promoter and downstream of eGFP to avoid transcription read-through from vector promoters into the eGFP gene and read-through from the *gut* promoter into vector sequences (see Fig. 1). The *ganA* gene was amplified by polymerase chain reactions (PCR) from chromosomal DNA of *B. subtilis* with oligonucleotides s9872 and s9873 (Table 2) and inserted between the *Bam*HI and *Bsr*GI site of pJOE8386.1 to get pHWG1132. Hereby eGFP was deleted and the *ganA* gene without the translation start codon was fused to the first six codons of *gsiB* and the two codons provided by the *Bam*HI site (Fig. 1).

**Table 2:** Oligonucleotides used

Number	Sequence^a^	Purpose
S9732N	5′-aaggccaacgaggccAGCAGATGCTGCAGC	Construction of pJOE9898.1 flanking sequences
S9733N	5′-aaggccagtctggccGATCAGCTTAATTGCGCTG	
S9734N	5′-aaggcctaaatggccGACAGAAGCGGTGTGAC	
S9735N	5′-aaggccttattggccGTCGGAGCAATGACGTTTA	
S12531	5′-tacgGAATCCCTCACATCTGAAAT	Construction of pJOE9898.1 spacer
S12532	5′-aaacATTTCAGATGTGAGGGATTC	
S12618	5′-GAAGCACTGATTCTATCACC	Colony PCR to prove the deletion by pJOE9898.1
S12619	5′-CATCCAGCCATTCAAATTGA	
S9703N	5′-aaggccaacgaggccACTTGAAGGCCGTAACATT	Construction of pJOE9899.1, flanking sequences
S9704N	5′-aaggccagtctggccTGCTTAGCGGGCAGT	
S9705N	5′-aaggcctaaatggccTGTTAATGATGCAAGGGCT	
S9706N	5′-aaggccttattggccCGGCGTCCAATCGTTTTAC	
S12533	5′-tacgAGAATTAGAAGATAAAATTG	Construction of pJOE9899.1, spacer
S12534	5′-aaacCAATTTTATCTTCTAATTCT	
S12620	5′-CAGAGGACTGTACCATGAT	Colony PCR to prove the deletion by pJOE9899.1
S12619	5′-CATCCAGCCATTCAAATTGA	
S9724N	5′-aaggccaacgaggccCAGGAAGCTGTTGAATCT	Construction of pJOE9918.1, flanking sequences
S9725N	5′-aaggccagtctggccGCCCTTCTTTTTCTACTCTA	
S9726N	5′-aaggcctaaatggccGCTCATTTTCTTAAAAAGAATATC	
S9727N	5′-aaggccttattggccGGCGATAAAGAATTCGAAG	
S12529	5′-tacgGCAATCAGCTTCAGTCGAAA	Construction of pJOE9918.1, spacer
S12530	5′-aaacTTTCGACTGAAGCTGATTGC	
S12660	5′-CGGCCTGTTGAATATTAAAC	Colony PCR to prove the deletion by pJOE9918.1
S12619	5′-CATCCAGCCATTCAAATTGA	
S9707N	5′-aaggccaacgaggccCATGATGTTTGTCCCTAG	Construction of pJOE9957.1, flanking sequences
S9708N	5′-aaggccagtctggccGTTTCATACTGTGCTAAAGA	
S9709N	5′-aaggcctaaatggccGTACCCTATAGTTATTTAAATCC	
S9710N	5′-aaggccttattggccCCCAAATCGAACACGGTTCA	
S12649	5′-tacgATGTCAGCAATACACACAAA	Construction of pJOE9957.1, spacer
S12650	5′-aaacTTTGTGTGTATTGCTGACAT	
S12715	5′-GAATGCATCCACAGCAGG	Colony PCR to prove the deletion by pJOE9957.1
S12716	5′-GGCATAAATCACAGTGGCA	
S9795	5′-aaggccaacgaggccGGGCTTGTCTTTATCGTG	Construction of pJOE9974.1, flanking sequences
S9796	5′-aaggccagtctggccGATGTGAAGACTGGAG	
S9797	5′-aaggcctaaatggccGCCTGGCTTTGATTACGTG	
S9798	5′-aaggccttattggccGTGCTCTCCGATAATATGC	
S12673	5′-tacgGCTTATATCTATAAACATGA	Construction of pJOE9974.1, spacer
S12674	5′-aaacTCATGTTTATAGATATAAGC	
S12740	5′-CGGTAAGTCCCGTCTAGC	Colony PCR to prove the deletion by pJOE9974.1
S12741	5′-GGGAAGCGTTCACAGTTTC	
S12799	5′-aaggccagactggccTAAAAGTACAGTGCCGCTG	Amplification of *Pgut*-*ganA*
S12800	5′-aaggccatttaggccCCGAAAAGTGCCACCTG	
S10494	5′-GACCTCAAAAAGGTCTTTA	DNA sequencing of inserts in pJOE8999.1
S11222	5′-CACGCATTGATTGAGTCAG	

^a^Small letters: nucleotides added for cloning

All CRISPR/Cas9 vectors for insertion of *Pgut*-*ganA* into the *B. subtilis* chromosome were constructed in the same way as will be demonstrated for pJOE9898.1. A 20 nucleotide spacer sequence for guiding the Cas9 to the target site was selected from gene *spoIIA* using the software CCTop (Stemmer et al. 2015). The two complementary oligonucleotides were modified by adding four bases to the 5′ ends to fit to the single-strand ends generated by cutting pJOE8999.1 by *Bsa*I (see oligonucleotides s12531/s12532, Table 2). The oligonucleotides were annealed by heating to 95 °C for 5 min, and slowly cooling down to room temperature and ligated to *Bsa*I cut vector pJOE8999.1. Colonies with cloned spacers were identified by blue-white screening on LB plates supplemented with ampicillin, IPTG and X-Gal. Plasmid DNA from white colonies were isolated and sequenced with the primer s11222 (see Table 2). The verified new plasmid was cut with *Sma*I and *Sfi*I and three *Sfi*I-fragments were ligated with the vector DNA at the same time, consisting of two PCR fragments from the flanking regions obtained with primers s9732N/s9733N and s9734N/s9735N and the expression cassette obtained by PCR from pHWG1132 with primers s12799/s12800. The ligation was done overnight at room temperature in 10 µl volume with 100 ng vector DNA, 20 ng of each flanking fragments and 40 ng of the expression cassette fragment. The different *Sfi*I sites at the ends of the various fragments and vector allowed the ordered cloning of the three fragments. The other plasmids (pJOE9899.2, pJOE9918.5, pJOE9957.1 and pJOE9974.2) were constructed in the same way using primers shown in Table 2.

### DNA transformation

Standard molecular techniques, such as *E. coli* transformation, were carried out as described by Sambrook and Russell (2001). Induction of competence and transformation of *B. subtilis* Reg19 was performed as described before (Rahmer et al. 2015). In brief, an overnight culture of REG19 cultivated in LB, was diluted to an optical density of 0.05 OD_600_ in 9 ml LB and incubated in a 100 ml shake flask at 37 °C for 1.5 h at 200 rpm. Competence was induced by adding 1 ml LB containing 5% (w/v) mannitol and further incubation for another 1.5 h. The cells were washed by centrifugation (Sorvall Megafuge, 4500 rpm, room temperature) and resuspended in the same volume of LB. To 1 ml culture about 500 ng plasmid DNA was added and the cells incubated at 37 °C for 1 h in case of pHWG1132 and in case of pJOE8999 and derivatives at 30 °C for 1.5 h. Finally, the cells were plated on LB agar plates containing 5 µg/ml kanamycin and incubated overnight at 37 °C or 30 °C depending on the plasmid used.

### DNA techniques

The desired DNA fragments were amplified by PCR utilizing Q5 DNA polymerase (New England BioLabs^®^, Frankfurt am Main, Germany) on a PTC-200 Peltier Thermal Cycler (MJ Research). Oligonucleotides obtained from Eurofins MWG and used in this study are listed in Table 1. Commercial kits including DNeasy^®^ Blood & Tissue Kit (Cat. #69506; Qiagen, Hilden, Germany) for chromosomal DNA extraction, “innuPREP Plasmid mini Kit” (Analytic Jena AG, Jena, Germany) for plasmid isolation and “NucleoSpin^®^ Gel and PCR Clean-up” kit (Machery-Nagel, Düren, Germany) for PCR fragment purification were applied through-out this study. Restriction enzymes were provided by New England BioLabs^®^ (Frankfurt am Main, Germany). T4 DNA Ligase was purchased from Thermo Fisher Scientific Inc. (Karlsruhe, Germany). Plasmid DNA was sequenced by GATC Biotech (Konstanz, Germany).

### Insertion of *P*_*gut*_-*ganA* into the *B. subtilis* chromosome and curing of the plasmids

Plasmid DNA (about 500 ng in 10 µl) was added to competence induced REG19 (1 ml) and the cells were incubated at 30 °C for 1.5 h on a roller drum and then plated on LB agar supplemented with kanamycin at 30 °C for 2 days. Colonies were streaked out on LB agar plates with kanamycin and 0.5% mannose to induce *cas9* expression. For plasmid curing, cells were then streaked out on LB agar plates at 50 °C and after growth streaked out again to single colonies on LB agar plates at 42 °C. Finally, cells were tested at 30 °C on LB plates supplemented with kanamycin for loss of the plasmid and by colony PCR for correct insertion/deletions within the chromosome (see Table 2).

### Measurement of β-galactosidase activity

After the growth of the cells for 16 h in LB media containing either glucitol or glucose, a volume containing 10 OD_600_ of cells (approximately 1 × 10^10^ cells) were harvested by centrifugation and the cell pellet was resuspended in 1 ml 0.1 M potassium phosphate buffer, pH 6.5. Crude cell extract was prepared using ultrasonic sound (3 × 45 s, 50% duty cycle; Heat Systems-Ultrasonics, Inc. model W-385 sonicator, Farmingdale, New York, USA). After centrifugation, the supernatant of the lysate (cleared lysate) was used for the enzyme assay. The activity of β-galactosidase was determined by measuring the rate of *p*-nitrophenyl β-d-galactopyranoside (pNP-βGal) hydrolysis as described earlier (Watzlawick et al. 2016). One unit of the enzyme activity was defined as the release of 1 µmol of pNP per minute at 37 °C. Protein concentration was determined by the method of Bradford (1976) with bovine serum albumin as a standard. SDS-PAGE was done according to the method of Lämmli (1970).

## Results

### Construction of the expression vector pHWG1132 and five different CRISPR/Cas9 vectors for integration of *ganA* into the *B. subtilis* chromosome

The expression vector pHWG1132 (Fig. 1) is a shuttle vector consisting of the replication origin and the kanamycin resistance gene of pUB110 and the replication origin and the ampicillin resistance gene of pIC20HE. In addition, it contains the expression cassette of *ganA*, where the *ganA* gene of *B. subtilis* was under control of the *B. subtilis* glucitol promoter (*P*_*gut*_) and flanked by rho-independent transcriptional terminators upstream of *P*_*gut*_ and downstream of *ganA*. Between *gut* promoter and *ganA* the translation initiation sequence of *gsiB* was inserted for efficient translation of *ganA*. With this plasmid the *B. subtilis* strain REG19, containing a second copy each of the competence genes *comK*–*comS* genes under the mannitol promoter, was transformed as described (Rahmer et al. 2015).

**Fig. 2:**
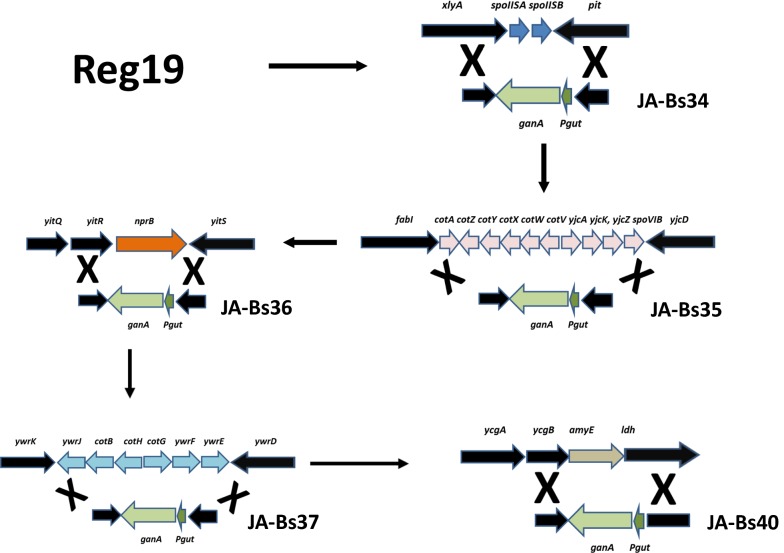
Diagram showing the various sites of integration of the *P*_*gut*_-*ganA* cassette, the genes deleted during the integration and genes enabling integration via homologous recombination

For integration of the *P*_*gut*_-*ganA* expression cassette into *B. subtilis* chromosome five regions within the chromosome were selected (Barbe et al. 2009) which were supposed to allow integration of *ganA* as well deletion of genes at the integration sites without affecting growth of the strains (Zhu and Stülke 2018). In strain JA-Bs34 the *P*_*gut*_-*ganA* cassette was integrated between *xylA* and *pit*. Hereby the genes *spoIISA* and *spoIISB* were deleted (see Fig. 2). In JA-Bs35 the expression cassette was integrated between *fabI* and *yjcD* deleting 10 genes from *cotA* to *spoVIF*. In JA-Bs36 the cassette was integrated between *yitR* and *yitS* deleting the protease encoding gene *nprB*. JA-Bs37 had the expression cassette integrated between *ywrK* and *ywrD* producing a deletion of six genes from *ywrJ* to *ywrE* and finally in JA-Bs40 the expression cassette was integrated between *ycgB* and *ldh* replacing *amyE*. The CRISPR/Cas9 vectors were constructed in the following way. First, a spacer sequence was cloned into the CRISPR/Cas9 vector pJOE8999, targeting Cas9 to the region which should be deleted during integration. The *P*_*gut*_-*ganA* sequence from pHWG1132 was amplified by PCR with oligonucleotides containing *Sfi*I sites at the ends. In addition 700–800 bp sequences flanking the insertion sites and having compatible *Sfi*I sites to the vector and the *P*_*gut*_-*ganA* cassette were amplified by PCR as well and the vector and the three PCR fragments were ligated in one step. The CRISPR/Cas9 vectors pJOE9898.1, pJOE9899.2, pJOE9918.5, pJOE9957.1 and pJOE9974.2 generated in this way were used to transform REG19, one after the other to insert the *P*_*gut*_-*ganA* expression cassette five times into the *B. subtilis* chromosome (Fig. 2).

### Production of GanA

**Fig. 3:**
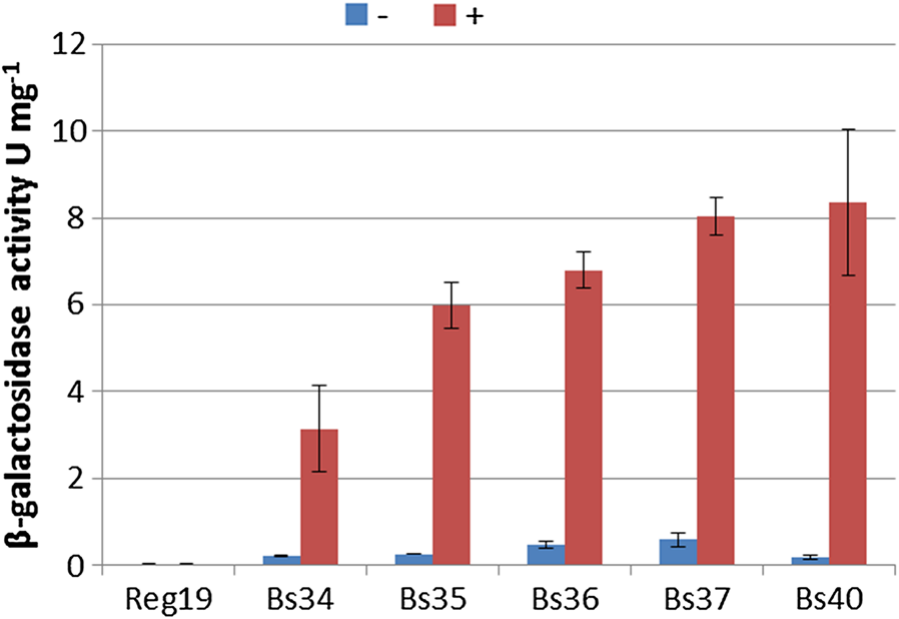
GanA ß-galactosidase activity in cleared crude extracts from *B. subtilis* containing none (REG19), 1 copy (JA-Bs34), 2 copies (JA-Bs35), 3 copies (JA-Bs36), 4 copies (JA-Bs37) and 5 copies (JA-Bs40) of the *P*_*gut*_-*ganA* cassette. The cells were grown in LB with either 0.5% glucitol (+) or 0.5% glucose (−) and after 16 h crude cell extract was prepared for determining β-galactosidase activity and protein concentration. The values are the mean of three different experiments

**Fig. 4:**
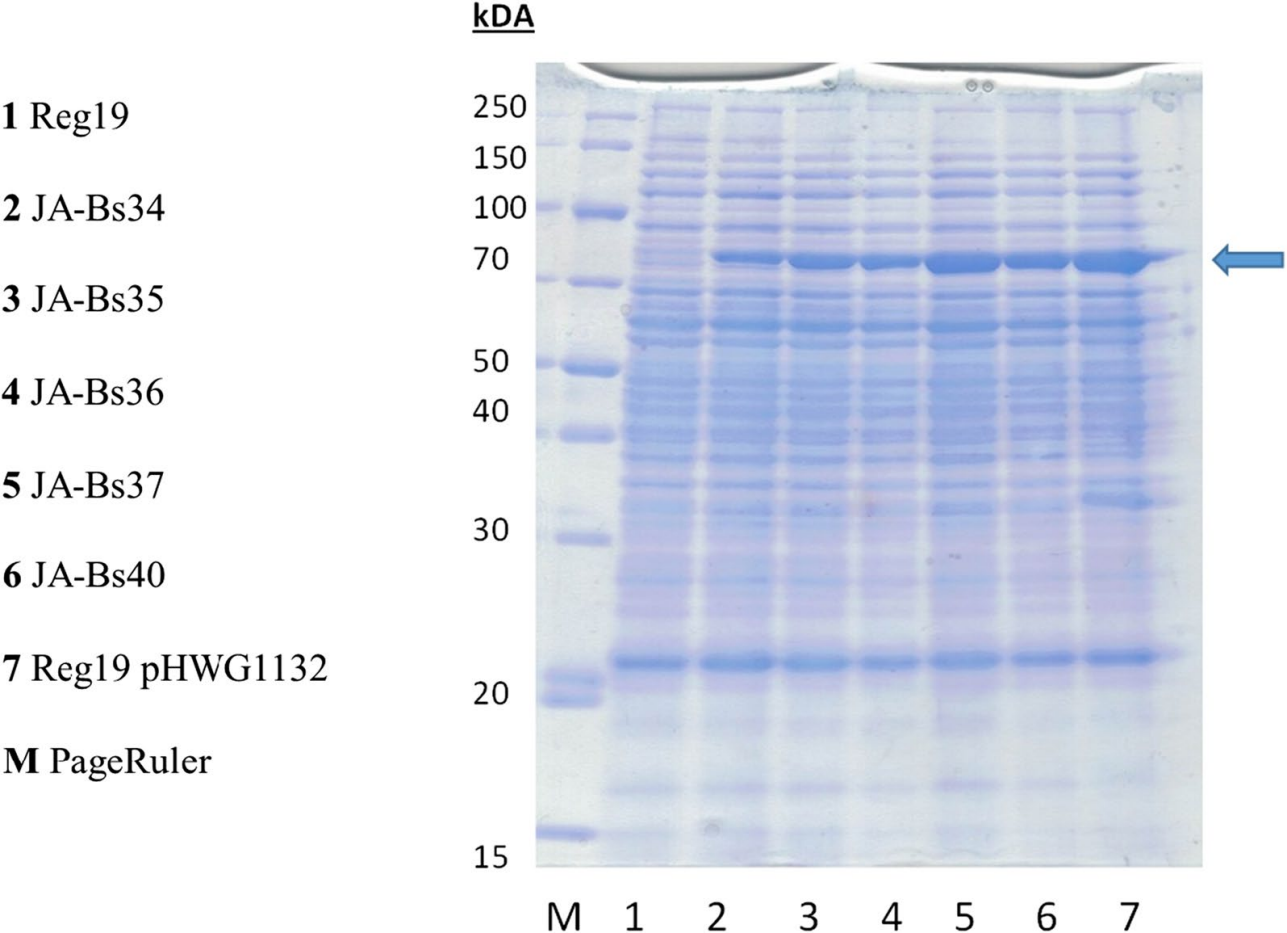
SDS-PAGE showing the protein crude extracts from REG19, JA-Bs34 to JA-Bs40 and from REG19 carrying the plasmid pHWG1132. Crude extracts were prepared from cells induced by glucitol for 16 h, cleared by centrifugation and the amount of 15 μg cleared crude extract proteins were applied on the SDS-gel in each line. The GanA protein of 79.8 kDa is indicated by the arrow

The GanA activity was determined with the synthetic substrate *p*-nitrophenyl-β-d-galactopyranoside (pNP-βGal). Cells of a stationary overnight culture in LB were diluted to an optical density of 0.05 OD_600_ in LB with 0.5% glucitol and grown in baffled shaking flasks at 200 rpm and 37 °C for 16 h. The cells were harvested by centrifugation, resuspended in 0.1 M sodium phosphate buffer pH 6.5 and lysed by ultrasonication. The GanA activity of the cleared crude cell extract was determined with pNP-βGal as substrate. The specific β-galactosidase activities in units per mg protein obtained are shown in Fig. 3. The strain REG19 contains already one copy of *ganA* within the *gan*-operon. This gene is only transcribed from its native *gan* promoter by induction with galactan (Watzlawick et al. 2016). Therefore, this copy in REG19 produced nearly no activity under the used conditions in contrast to the second copy in JA-Bs34, now under the *gut* promoter. In the strain JA-Bs35 this activity was duplicated. In JA-Bs36, JA-Bs37 and JA-Bs40 with 3, 4 and 5 copies respectively, the β-galactosidase activity increased as well but at a lower rate as expected. The strain JA-Bs40 showed an activity in the same range (8.36 ± 1.19 U/mg) as it was determined with glucitol-induced REG19/pHWG1132 cells (9.83 ± 1.15 U/mg). In the strains grown with glucose the β-galactosidase activity was about 20-fold lower as expected from an inducible promoter underlying in addition catabolite repression (Görke and Stülke 2008). The SDS-Page with the crude extracts from the various strains (Fig. 4) showed an increasingly prominent protein band of 79.8 kDa which corresponds to the molecular weight of GanA.

### Comparing the stability of JA-Bs40 and REG19 pHWG1132 concerning GanA production

**Fig. 5:**
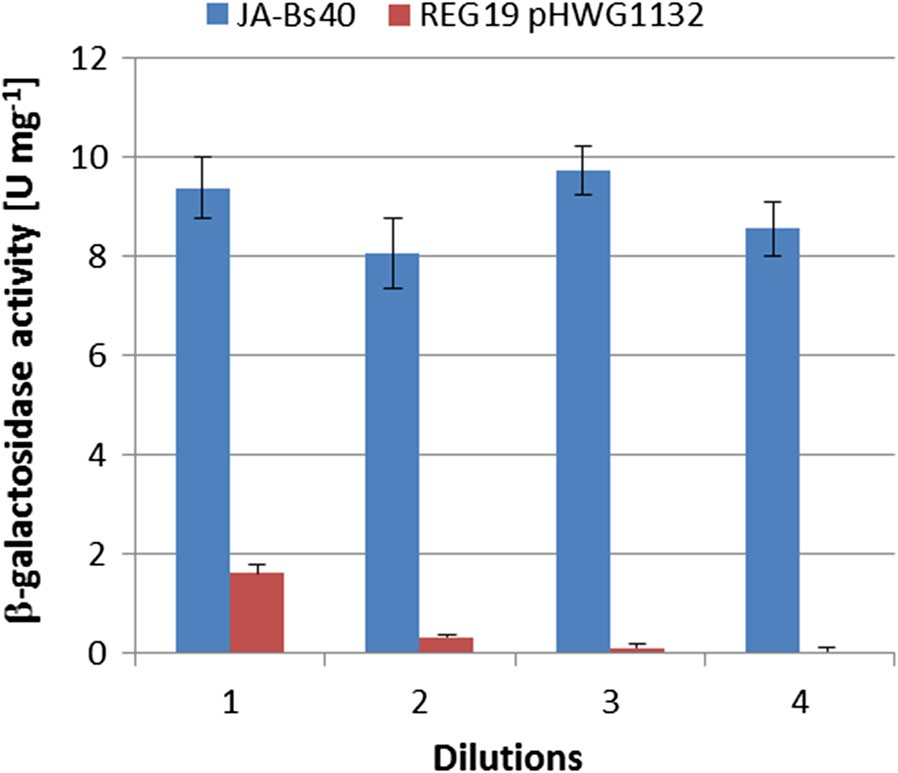
GanA ß-galactosidase activities in crude extracts of cells from JA-Bs40 and REG19 pHWG1132 diluted 1000-fold and grown to stationary phase repeatedly without antibiotics in LB with 0.5% glucitol. The values are the mean of three different experiments

The stability of strain JA-Bs40 and REG19/pHWG1132 in the production of GanA under *ganA* induced conditions (adding of glucitol) and without antibiotic selection of the plasmid was compared. Overnight cultures of both strains in LB with 0.5% glucose and in case of REG19/pHWG1132 with glucose and kanamycin were diluted 1:1000 in LB with 0.5% glucitol. The cells were grown in shaking flasks at 37 °C to stationary phase and diluted again 1000-fold. This was repeated three times and each time when the stationary phase was reached, the β-galactosidase activity was determined. The results are presented in Fig. 5. The strain JA-Bs40 continuously produced about the same amount of GanA over the time. In contrast, REG19/pHWG1132 showed only about 20% of GanA activity even after the first round of growth to stationary phase corresponding to about nine generations without antibiotic supplementation. The GanA activity decreased continuously from dilution to dilution and was nearly not detectable at the end of the last round of growth. When the cells from this last round were plated to single colonies on LB agar plates and tested for kanamycin resistance, none of the 70 colonies checked were able to grow on LB agar plates supplemented with kanamycin. This demonstrated the high frequency of plasmid loss.

## Discussion

The use of multi-copy expression vectors for heterologous protein production is straightforward and needs only one cloning step and one transformation procedure to get a suitable production strain. The disadvantage of these vector systems are its instability (Fleming and Patching 1994; Shoham and Demain 1991) as it was demonstrated here with the pUB110 derivative pHWG1132 for production of GanA. Under induced expression conditions it needed the presence of an appropriate antibiotic to select for cells still carrying the plasmids and producing GanA in high amounts. This is in contrast to the strains with multiple integrations of expression cassettes. They are tedious to construct but need no longer antibiotics. The use of the CRISPR/Cas9 system is especially useful since integration is selected due to the double-strand cut of the target site and repair by homologous recombination. With traditional methods, integration is selected via an antibiotic resistance gene which has to be removed in a second procedure (Bloor and Cranenburgh 2006; Sanchez et al. 2007; Sanchis et al. 1997). Such methods usually leave behind recognition sites for recombinases causing maybe problems in the next rounds of insertions.

For successful insertions into the chromosome, the expression cassette has to be flanked by sequences of several hundred base pairs length obtained from both sides of the target region. Such insertion cassettes can be constructed in a traditional way by cloning first the expression cassette into a polylinker sequence of a vector and then adding upstream and downstream sequences from the target. As shown in this report, the procedure can be reduced to one cloning step by using the *Sfi*I sites in the CRISPR/Cas9 vector pJOE8999 and the *Sfi*I sites added to the fragments by PCR. They allowed an ordered ligation of the PCR fragments with the vector in one step. Alternatives would by the alignment of vector and fragments with overlapping ends by Gibson assembly or by PCR.

The multiple gene integration procedure can also be used to remove unwanted properties of the strain like proteases (Zhang et al. 2019), prophages, antibiotic biosynthesis or sporulation. Even the deletion of the many putative genes of unknown function might be of advantage by reducing the metabolic burden of the cells (Manabe et al. 2012; Morimoto et al. 2008).

How many genes have to be integrated into the chromosome to get a maximal yield of protein depends on many parameters like promoter strength, translation efficiency, mRNA and protein stability etc. Locations nearer to the replication origin increases the average copy number in replicating cells and transcription in the same orientation as the replication fork moves on the chromosome avoids collisions with the replication machinery (Jeong et al. 2018; Sauer et al. 2016).

Taking in account the specific activity of a purified GanA protein of 80 U/mg with pNP-βGal as substrate (Watzlawick et al. 2016) the productivity of GanA with JA-Bs40 and Reg19/pHWG1132 is about 10% of their total soluble cell protein. This yield was also judged by SDS-PAGE of soluble GanA in cleared crude extracts. SDS-PAGE analysis of the cell pellet fraction showed no presence of an unsoluble GanA protein band. The production of soluble target proteins in *Bacillus* up to 30% of the cell proteins were reported by using other expression systems for the respective genes (Phan et al. 2015). In case of the obtained results during the expression of the *ganA* gene in this study, it is still not clear what limits *ganA* expression to get similar high amounts. It certainly is not the copy number of the expression cassettes since pUB110 is present at 48 copies per cell (Leonhardt 1990). Since *P*_*gut*_ needs the activator protein GutR that is located as a single copy on the chromosome, one could speculate that there is not enough activator produced. In consequence, the cloning of the *gutR* gene into the expression cassettes might lead to a further increase of GanA production.

In summary, the use of the CRISPR/Cas9 vectors to construct stable production strains for overproduction of enzymes and other proteins by multiple gene integration is a valuable, fast and easy to handle system. It avoids the presence of an antibiotic resistance gene and allows at the same time the improvement of the production strains by removal of unwanted properties.

## Data Availability

Please turn to the authors for all requests.
